# Molecular Mechanisms Responsible for Mesenchymal Stem Cell-Dependent Attenuation of Tear Hyperosmolarity and Immune Cell-Driven Inflammation in the Eyes of Patients with Dry Eye Disease

**DOI:** 10.3390/diseases12110269

**Published:** 2024-10-26

**Authors:** Carl Randall Harrell, Valentin Djonov, Ana Volarevic, Aleksandar Arsenijevic, Vladislav Volarevic

**Affiliations:** 1 Regenerative Processing Plant, LLC, 34176 US Highway 19 N, Palm Harbor, FL 34684, USA; drharrell@regenerativeplant.org; 2 Institute of Anatomy, University of Bern, Baltzerstrasse 2, 3012 Bern, Switzerland; valentin.djonov@unibe.ch; 3 Department of Psychology, Center for Research on Harmful Effects of Biological and Chemical Hazards, Faculty of Medical Sciences, University of Kragujevac, 69 Svetozara Markovica Street, 34000 Kragujevac, Serbia; ana.volarevic@fmn.kg.ac.rs; 4 Departments of Genetics, Microbiology and Immunology, Center for Research on Harmful Effects of Biological and Chemical Hazards, Faculty of Medical Sciences, University of Kragujevac, 69 Svetozara Markovica Street, 34000 Kragujevac, Serbia; salvatoredjulijano@gmail.com; 5 Faculty of Pharmacy Novi Sad, Trg Mladenaca 5, 21000 Novi Sad, Serbia

**Keywords:** mesenchymal stem cells, dry eye disease, tear hyperosmolarity, immune cell-driven inflammation, dysfunction of ion channels

## Abstract

Background: Dry eye disease (DED) is a chronic condition characterized by a decrease in tear production or an increase in tear evaporation, leading to inflammation and damage of the ocular surface. Dysfunction of ion channels, tear hyperosmolarity and immune cell-driven inflammation create a vicious circle responsible for the pathological changes in the eyes of DED patients. Mesenchymal stem cells (MSCs) are adult, rapidly proliferating stem cells that produce a large number of immunoregulatory, angiomodulatory, and growth factors that efficiently reduce tear hyperosmolarity-induced pathological changes, inhibit harmful immune response, and provide trophic support to the injured corneal and conjuctival epithelial cells, goblet cells and acinar cells in lacrimal glands of DED patients. Methods: An extensive research in the literature was implemented in order to elucidate the role of MSCs in the attenuation of tear hyperosmolarity and eye inflammation in patients suffering from DED. Results: Findings obtained in preclinical and pilot clinical studies demonstrated that MSCs reduced tear hyperomsolaity-induced pathological changes and suppressed immune cell-driven eye inflammation. Additionally, MSC-based therapy managed to successfully address the most severe DED-related conditions and complications. Conclusions: MSCs should be considered as potentially new therapeutic agents for the treatment of severe DED.

## 1. Introduction

Dry eye disease (DED) is a multifactorial condition characterized by a loss of homeostasis in the tear film, leading to ocular discomfort and potential damage to the ocular surface. Key clinical features include persistent dryness, a gritty or sandy sensation in the eyes, and fluctuating visual acuity, often exacerbated by environmental factors such as wind or prolonged screen time. Patients may also experience redness and increased sensitivity to light, along with excessive tearing that, paradoxically, occurs as a response to irritation [1].

The severity of DED is defined through a combination of clinical signs and patient-reported symptoms, classified into mild, moderate, and severe categories. In mild DED, patients may report occasional dryness or a slight burning sensation, with minimal signs observed during an eye examination, such as normal tear break-up time and no significant corneal staining. Moderate DED is characterized by more frequent symptoms, including persistent dryness, irritation, and fluctuating vision, combined with signs like reduced tear production or mild corneal staining. In severe DED, patients experience chronic discomfort that significantly impacts daily life, often reporting sensations of grittiness or excessive tearing. Clinical signs reveal notable corneal damage, marked tear deficiency, and possibly inflammation of the conjunctiva, making it crucial for healthcare providers to assess both subjective experiences and objective findings when determining the severity and appropriate management strategies for DED [2].

Current therapeutic approaches for severe DED encounter various limitations and challenges. Artificial tears, commonly used to manage severe DED, have notable limitations that can hinder their effectiveness. Many of them contain preservatives which can exacerbate ocular surface inflammation with prolonged use, ultimately worsening symptoms. Even preservative-free artificial tears can be inadequate since they typically need to be applied frequently to provide relief, leading to patient non-compliance. While artificial tears may provide temporary moisture, they do not address the underlying causes of dry eye, such as inflammation or meibomian gland dysfunction (MGD). Consequently, for many patients suffering from severe DED, artificial tears could serve just as a short-term solution rather than a comprehensive treatment strategy. Although anti-inflammatory medications such as corticosteroids can provide long-term relief, they carry risks of side effects, including increased intraocular pressure and cataract formation, making them unsuitable for chronic management. Moreover, therapies like punctal plugs, which aim to retain tears on the ocular surface, may not fully alleviate symptoms for all patients. Some individuals find them uncomfortable or experience complications like infection or displacement. Surgical interventions, such as salivary gland duct occlusion, may offer benefits, but are invasive and not universally applicable, posing risks of complications. Lastly, novel treatments like autologous serum tears can be effective but require a complicated preparation process and are often not widely accessible, limiting their use. These limitations highlight the need for more targeted, personalized therapies that address the underlying mechanisms of severe DED while minimizing side effects and improving overall patient quality of life [3].

Various factors such as aging, eye injuries, MGD, surgical interventions in the eye, inadequate nutrition, environmental allergens, and chemical exposures can disrupt ion channel proteins and trigger harmful immune responses in the eye, contributing to the onset and worsening of severe DED [4]. The resulting tear hyperosmolarity, stemming from changes in ion channels, coupled with ocular inflammation due to increased immune cell activation and excessive inflammatory cytokine production, creates a vicious cycle that exacerbates all key DED-related signs and symptoms, including ocular discomfort, pain, and vision problems. Therefore, a deeper understanding of the DED pathogenesis, especially the contributions of inflammation, tear film instability, and MGD, can greatly improve treatment efficacy. This understanding facilitates the creation of customized treatment plans that address the unique underlying factors for each patient, ultimately improving outcomes and quality of life. By bridging the gap between pathophysiology and therapy, new and more effective therapeutic agents could be offered to patients suffering from severe DED [5].

Accordingly, in this review article, we summarized current knowledge about the molecular mechanisms that are responsible for the inflammation-induced modification of ion channels leading to the generation of tear hyperosmolarity that elicits immune cell dysfunction in the eyes of DED patients. We also emphasized the therapeutic potential of mesenchymal stem cells (MSCs) and their secretome, which are capable of stabilizing the tear film, suppressing a detrimental immune response, and regenerating injured meibomian glands, opening new avenues for addressing tear hyperosmolarity-dependent immune cell-driven inflammation in the eyes of DED patients. An extensive literature review was carried out in October 2024 across several databases (MEDLINE, EMBASE, Google Scholar), from 1990 to the present. Keywords used in the selection were as follows: ”dry eye disease”, “mesenchymal stem cells”, “tear hyperosmolarity”, “ion-channels”, “meiboman gland dysfunction”, “signaling pathways”, “inflammation”, “immune cells”, “immunosuppression”, “immunoregulation”, and “tissue repair and regeneration”. All journals were considered and an initial search retrieved 317 articles. The abstracts of all these articles were subsequently reviewed by two of the authors (CRH and VV) independently to check their relevance to the subject of this manuscript. A total number of 84 eligible studies, which delineated molecular and cellular mechanisms that were involved in the dysfunction of ion channels, the pathogenesis of immune cell-driven eye injury and inflammation, and the MSC-dependent attenuation of tear hyperosmolarity-induced pathological changes in the eyes of DED patients, were analyzed in detail and their findings were summarized in this review.

## 2. Dysfunction of Ion Channels, Tear Hyperosmolarity, and Immune Cell-Driven Inflammation: A Vicious Circle Responsible for the Pathological Changes in the Eyes of DED Patients

Ion channels such as transient receptor potential ankyrin 1 (TRPA1) and transient receptor potential melastatin 8 (TRPM8) are essential for cellular ion balance, playing an important role in maintaining ocular surface health. TRPA1 specifically regulates calcium ion influx across the cell membrane, which governs sensation in the face and eyes. Studies in DED animal models revealed that activating TRPA1 results in decreased tear production, increased blinking, and eye-wiping behavior. TRPM8, a nonselective cation channel, is critical in DED development as it responds to tear hyperosmolarity caused by evaporation. When activated, it raises intracellular calcium levels, stimulating tear secretion from lacrimal glands. Reduced TRPM8 levels in the cornea correlate with decreased sensitivity and tear production, whereas increased TRPM8 expression enhances tear secretion without causing pain [6].

Importantly, TRPM8 negatively regulates the expression of TRPV1, an ion channel that is involved in pain perception and inflammation [6,7]. When activated, TRPV1 allows large cations to enter the cell, triggering a cascade of events including calcium influx, channel relocation, and cytokine production. In the context of DED, TRPV1 activation in CECs triggers the release of inflammatory factors like interleukin (IL)-6 and IL-8, which work alongside tumor necrosis factor alpha (TNF-α) and IL-1β to enhance inflammatory responses. Clinical trials using small interfering RNA (siRNA) to inhibit TRPV1 in DED patients have yielded promising outcomes, demonstrating reduced pain and improved clinical scores, highlighting TRPV1 as a potential therapeutic target. Notably, TRPM8 stimulation inhibits TRPV1 activation in eye tissues, indicating its role in controlling inflammation in the eyes of DED patients. Both TRPM8 and TRPV1 interact with sodium and potassium channels, affecting their expression levels. Inhibiting TRPM8 in DED models decreases mRNA levels of sodium channels Nav1.7 and Nav1.8, while TRPV1 blockade results in similar reductions. TRPV1 also interacts with purinergic receptors (P2X and P2Y2), which are involved in tear secretion and glycoprotein release by goblet cells, suggesting their potential role in DED development and progression [7].

**Figure 1:**
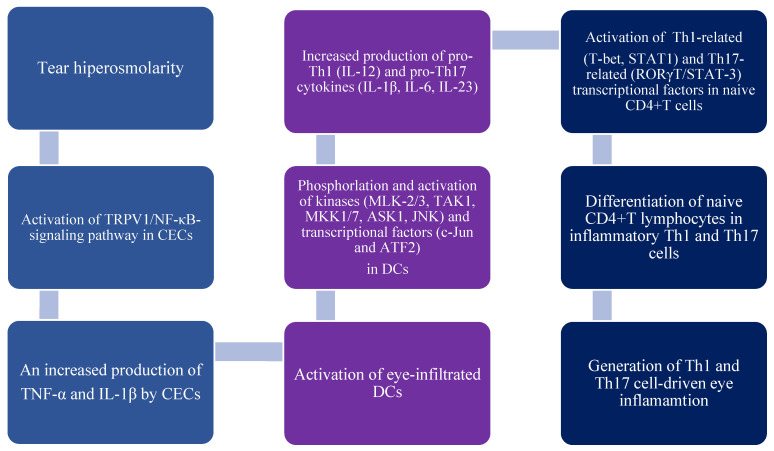
Tear hyperosmolarity-induced generation of Th1 and Th17 cell-driven eye inflammation. Hyperosmolarity activates the transient receptor potential vanilloid 1 (TRPV1)-dependent signaling pathway in corneal epithelial cells (CECs), which leads to the activation of the transcription factor nuclear factor kappa B (NF-κB). This activation results in an increased expression of genes for tumor necrosis factor alpha (TNF-α) and interleukin (IL)-1β, subsequently enhancing the production of these inflammatory cytokines. IL-1β and TNF-α stimulate various kinases, including mixed lineage kinases (MLK)-2/-3, transforming growth factor β-activated kinase 1 (TAK1), mitogen-activated protein kinase (MKK)-1, MKK7, apoptosis signal-regulating kinase 1 (ASK1), and Jun N-terminal kinase (JNK) in eye-infiltrating antigen-presenting dendritic cells (DCs). These kinases then phosphorylate and activate the transcription factors c-Jun and ATF2. Once activated, c-Jun and ATF2 translocate to the nucleus, where they enhance the expression of pro-Th1 (IL-12) and pro-Th17 cytokines (IL-1β, IL-6, IL-23) in DCs. By producing IL-12, DCs activate transcriptional factors T-box protein expressed in T cells (T-bet) and signal transducers and activators of transcription (STAT)-1 in naive CD4+ T lymphocytes, driving their differentiation into interferon-gamma (IFN-γ)-producing Th1 cells. Similarly, DC-derived IL-1β, IL-6, and IL-23 stimulate STAT-3- and RAR-related orphan receptor gamma T (RORγT) transcriptional factors in naive CD4+ T cells, promoting the development and proliferation of inflammatory CD4+ Th17 cells.

In line with these findings, alterations in the structure or function of TRPA1, TRPV1, and TRPM8 ion channels can induce hyperosmolarity of the tears, initiating a critical step in the development of potent inflammatory response in the eyes of DED patients [8]. Hyperosmolarity induces TRPV1-dependent activation of nuclear factor kappa B (NF-κB) in CECs, enhancing the production of IL-1β and TNF-α [Figure 1]. These inflammatory cytokines activate several kinases and transcriptional factors in eye-infiltrating DCs, promoting DC-dependent differentiation of naïve T cells in inflammatory Th1 and Th17 cells [8].

Th1 and Th17 lymphocytes play the most important pathogenic role in the progression of severe DED [9]. In the eyes of DED patients, Th1 cell-derived interferon-gamma (IFN-γ) and Th17 cell-sourced IL-17 and IL-22 induce the generation of inflammatory M1 phenotype in macrophages and N1 phenotype in neutrophils, crucially contributing to the generation of innate cell-driven inflammation [9,10]. Both N1 neutrophils and M1 macrophages are key sources of inflammatory mediators that lead to decreased mucin production, apoptosis of corneal epithelial cells (CECs) and conjunctival epithelial cells (ConECs), and the loss of goblet cells (GCs) in the inflamed eyes of DED patients [10]. Furthermore, M1 macrophages and N1 neutrophils release chemokines (C-X-C motif ligand (CXCL)-9/-10 and C-C motif ligand (CCL)-20) that attract circulating Th1 and Th17 cells into the inflamed eyes. Th1 and Th17 cell-sourced IFN-γ and IL-17 activate extrinsic and intrinsic apoptotic pathways and elevate the expression of matrix metalloproteinase (MMP)-3 and MMP-9 in CECs, resulting in the breakdown of the corneal epithelial barrier. These inflammatory cytokines also decrease tear production in lacrimal glands and induce apoptosis of meibocytes, crucially contributing to the development of MGD. Accordingly, elevated tear levels of IFN-γ and IL-17 heightened the risk of corneal ulcers and vision loss in DED patients and were associated with the onset of conjunctival epithelial squamous metaplasia [9,10].

Injured CECs, ConECs, GCs and activated eye-infiltrated immune cells secrete various inflammatory factors, including prostaglandins (PGE2), bradykinin, serotonin, TNF-α, IL-1β, IL-6 and IL-8, which bind to their respective receptors, activating protein kinase (PK)-A/B/C, mitogen-activated protein kinase (MAPK), and the Ca++/Calmodulin-dependent kinase I and II (CamKI/II)-driven intracellular cascades [8,9]. Activation of these signaling pathways results in the post-translational modification of ion channel proteins, affecting their structure and function [9]. Post-translational modifications of voltage-gated sodium channels (Navs) play a vital role in the development of tear hyperosmolarity-dependent DED [11]. Navs respond to depolarization of the transmembrane voltage, generating a quick, transient inward sodium current that drives the action potential’s rising phase. The voltage sensor domain of Navs contains charged residues that react to shifts in membrane potential. When transmembrane voltage changes, these charged regions move within the electric field, leading to conformational changes known as gating. Modifications involving charged groups in their intracellular, extracellular, or transmembrane areas affect the protein’s properties and functions. Beyond their direct electrostatic effects on gating, phosphorylation can also alter binding sites for regulatory proteins that influence Navs function. The inflammation-related post-translational modifications of Navs are mediated by various intracellular kinases [11]. The binding of PGE2 and bradykinin to their receptors primarily activates PKA and PKC kinases through adenylate cyclase and inositol 3-phosphate secondary messengers [9,10]. Additionally, the binding of TNF-α, IL-6, and neural growth factor (NGF) to their receptors engages ERK1/2 and p38 [10]. These signaling pathways can interact with one another. Increased kinase activation and the buildup of methylglyoxal enhance Nav1.7/Nav1.8 channel expression and function at the cell membrane, resulting in greater sodium influx and nociceptive neuronal hyperexcitability. Similarly, potassium channels may also be activated due to inflammation-driven post-translational modifications of their protein subunits [9,10,11].

Therefore, by inducing long-lasting structural and functional changes in untreated ion channels, a detrimental immune cell-driven inflammatory response causes tear hyperosmolarity and generates a vicious circle in the eyes that aggravates all DED-associated signs and symptoms [5,8,9,10]. Accordingly, therapeutic agents that could concurrently suppress Th1 and Th17 cell-driven eye inflammation and alleviate tear hyperosmolarity-dependent modulation of ion channels in the eyes of DED patients hold promise for the treatment of severe DED [4,12].

## 3. Therapeutic Potential of MSCs in the Treatment of DED

MSCs represent a versatile and promising avenue for the treatment of degenerative and inflammatory eye diseases due to their regenerative properties and ability to modulate detrimental immune responses in the eyes [13]. MSCs are self-renewable, rapidly proliferating, immunomodulatory stem cells that reside in multiple tissues, including bone marrow (BM), adipose tissue (AT), dental pulp (DP), umbilical cord (UC) and amniotic fluid (AF) [13,14]. MSCs are multipotent stromal cells that can spontaneously differentiate into a variety of cell types, including osteoblasts, chondrocytes, and adipocytes. However, in vitro, MSCs, which were grown under specific culture conditions, may differentiate in the cells of endodermal and ectodermal origin as well. Due to their enormous potential for differentiation, MSCs are considered promising candidates for treating various degenerative eye diseases, including age-related macular degeneration (AMD) and diabetic retinopathy. The therapeutic efficacy of MSCs in the treatment of these diseases has relied on the beneficial effects of MSC-sourced growth and pro-angiogenic factors (NGF, vascular endothelial growth factor (VEGF), epidermal growth factor (EGF) and angiopoietin-1 (Ang-1)), which slowed retinal degeneration, promoted vascular repair and enhanced the survival of retinal pigment epithelial cells [14].

**Figure 2:**
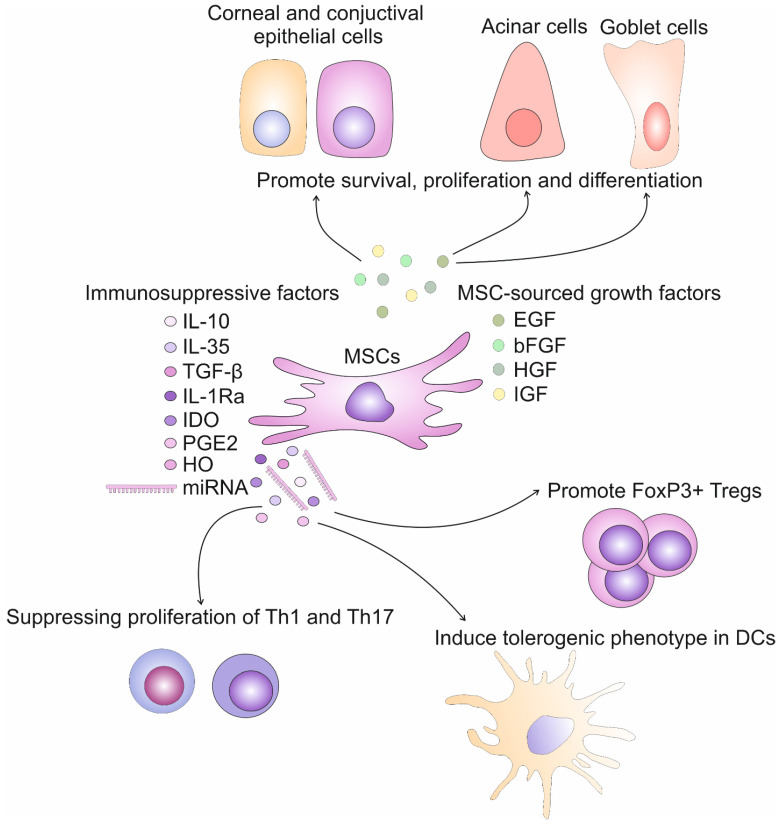
Immunoregulatory and regenerative properties of MSCs. MSCs produce immunosuppressive factors (IL-10, IL-35, TGF-β, IL-1Ra, IDO, PGE2, HO) and immunoregulatory miRNAs, which suppress proliferation of inflammatory Th1 and Th17 lymphocytes, induce tolerogenic phenotype in DCs and promote generation and expansion of FoxP3+ Tregs, creating immunosuppressive microenvironment in inflamed eyes of DED patients. Additionally, by transferring MSC-sourced growth factors (EGF, bFGF, HGF, IGF) into injured corneal and conjuctival epithelial cells, goblet cells, and acinar cells of lacrimal glands, MSC-Exos promote their survival, proliferation, and differentiation, importantly contributing to the alleviation of DED-related signs and symptoms.

An important characteristic of MSCs that differentiates them from other adult stem cells is their ability to suppress the detrimental immune response, attenuating the progression of inflammatory diseases [15]. MSCs are able to suppress the proliferation of activated immune cells in a juxtacrine (cell-to-cell contact)-dependent manner and a paracrine manner, through the activity of MSC-derived immunomodulatory factors [14,15]. MSC-sourced secretome is enriched with anti-inflammatory cytokines (IL-10, IL-35, transforming growth factor beta (TGF-β), IL-1 receptor antagonist (IL-1Ra)), immunosuppressive proteins (Indoleamine 2,3-dioxygenase (IDO), prostaglandin E2 (PGE2), heme oxygenase (HO)) and a large number of immunoregulatory microRNAs (miRNAs), which exert their effects by targeting key genes involved in immune cell activation, differentiation, and cytokine production, leading to the inhibition of pro-inflammatory pathways in activated immune cells [16]. These immunomodulatory molecules could also be delivered directly to target immune cells via MSC-derived exosomes (MSC-Exos), nanosized extracellular vesicles that possess a lipid bilayer membrane enabling the bypassing of all biological barriers in the body [17]. Additionally, by transferring MSC-sourced growth factors into injured CECs, ConECs, GCs, and acinar cells (ACs) of lacrimal glands, MSC-Exos can influence their survival, proliferation, and differentiation and promote enhanced repair and regeneration of damaged tissues [16,17] [Figure 2].

Importantly, MSCs are considered “immunoprivileged” cells due to their unique ability to evade recognition and attack by the immune system during allogeneic transplantation [18]. MSCs express low levels of major histocompatibility complex (MHC) class I molecules and lack MHC class II molecules, which are crucial for the immune recognition of transplanted cells and for the activation of allogeneic immune response [16]. Additionally, MSCs express co-inhibitory molecules (programmed death-ligand 1 (PD-L1) and B7-H4)), and, in an IDO-dependent manner, induce the generation and expansion of immunosuppressive T regulatory cells (Tregs), which play a key role in maintaining immune tolerance in transplanted grafts [18]. Accordingly, MSCs may be transplanted in MHC-mismatched recipients without the risk of rejection [18]. Since MSCs express a large number of chemokine receptors, after local or systemic administration, MSCs migrate to the site of eye injury or inflammation where they efficiently attenuate detrimental immune response and promote tissue repair and regeneration [19]. Their ability to mitigate tear hyperosmolarity-induced pathological changes, modulate phenotype and function of eye-infiltrated immune cells, and prevent apoptosis of injured CECs, ConECs, GCs, and ACs of lacrimal glands makes them promising candidates for the treatment of DED [13].

## 4. Molecular Mechanisms Responsible for MSC-Dependent Attenuation of Tear Hyperosmolarity in DED Patients

MSC-derived growth factors modulate the secretion of tear film components and promote the production of mucins, lipids, and aqueous fluid necessary for maintaining tear film stability [Table 1] [20]. By enhancing tear film quality and quantity, MSCs could efficiently mitigate tear hyperosmolarity and improve ocular surface lubrication and protection in the eyes of DED patients [21].

**Figure 3:**
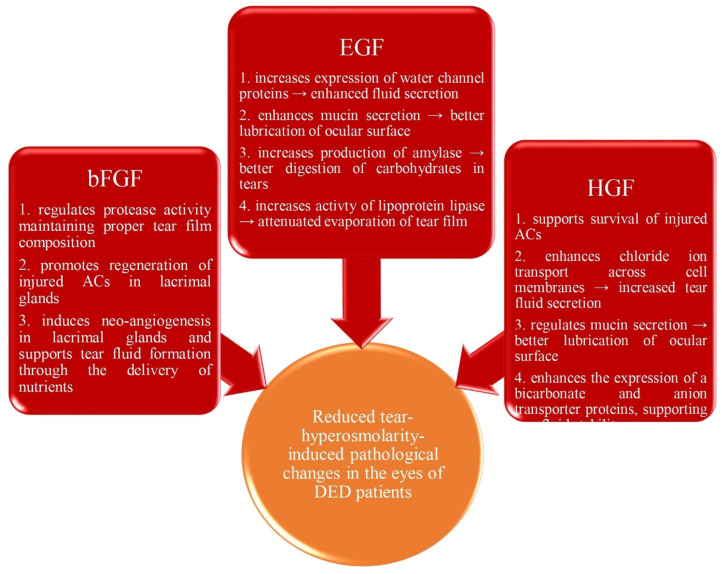
The role of MSC-sourced growth factors in the attenuation of tear hyperosmolarity. In the lacrimal glands, MSC-derived epidermal growth factor (EGF), basic fibroblast growth factor (bFGF), and hepatocyte growth factor (HGF) bind to their receptors on acinar cells (ACs) and activate various signaling pathways, which results in the increased expression of genes that control expression of water channel proteins, regulate synthesis of tear film components, and promote survival of injured cells, importantly contributing to the attenuation of tear hyperosmolarity-induced pathological changes in the eyes of patients with dry eye disease (DED).

In the lacrimal glands, MSC-derived EGF, basic fibroblast growth factor (bFGF), and hepatocyte growth factor (HGF) bind to their receptors on ACs and activate various signaling pathways, which results in the increased expression of genes that regulate the synthesis of tear film components [Figure 3] [21,22]. By increasing the expression of water channel proteins and by promoting the secretion of mucin and lipids, MSC-derived EGF enhances tear fluid secretion and lubrication of the ocular surface. Also, MSC-derived EGF increases the production and secretion of amylase, an enzyme that contributes to the digestion of carbohydrates in tears, ensuring proper tear film composition and function [21,22].

When MSC-derived bFGF binds to the FGF receptor (FGFR) in ACs of the lacrimal glands, it triggers the activation of several genes that play a role in enhancing tear fluid secretion [23,24]. Additionally, MSC-sourced bFGF can enhance the expression of the VEGFA gene, inducing neo-angiogenesis and vascular permeability [23,25]. An increased VEGFA expression promotes blood vessel formation in the lacrimal glands of DED patients, supporting tear fluid formation through the delivery of nutrients [25].

In ACs, MSC-derived HGF binds to its receptor, c-Met, leading to receptor dimerization and activation of downstream signaling PI3K/Akt and MAPK/ERK pathways [16,26]. Activation of c-Met by HGF enhances the survival and function of ACs, promoting tear fluid secretion and maintaining glandular integrity. Additionally, by binding to c-Met, MSC-sourced HGF up-regulates anterior gradient 2 (AGR2) gene expression, which regulates mucin synthesis in ACs, contributing to tear film stability and lubrication [26]. Similarly, MSC-derived HGF enhances the expression of bicarbonate and anion transporter proteins, aiding in tear fluid composition and stability [16,26].

**Table 1:** Beneficial effects of MSC-derived growth factors in the attenuation of tear hyperosmolarity in the eyes of DED patients.

MSC-Derived Factor	Activated Intracellular Signaling Pathways	Molecular Mechanisms Responsible for MSC-Based Effects	Beneficial Effects in the Eyes of DED Patients	Ref. No.
EGF	EGFR/MAPK/ERK/AQP4/AQP5	increased expression of AQP4 and AQP5 water channel proteins	enhanced tear fluid secretion	[21]
EGF	EGFR/MAPK/ERK/MUC5AC	up-regulated expression of MUC5AC protein	improved lubrication and protection of ocular surface	[21]
EGF	EGFR/MAPK/ERK/AMYL	increased production of amylase	improved tear film composition	[22]
EGF	EGFR/MAPK/ERK/LIPF	enhanced secretion of lipids	attenuated evaporation of tears	[22]
bFGF	bFGF/SPINK1	regulation of protease activity	improved tear film composition	[23]
bFGF	bFGF/PDGFA/VEGFA	increased neo-angiogenesis	improved delivery of nutrients to injured cells	[23,25]
HGF	c-Met/PI3K/CLCA1	enhanced chloride ion transport across cell membranes	enhanced tear fluid secretion	[26]
HGF	c-Met/PI3K/AGR2	increased mucin secretion	improved tear film stability and lubrication	[26]
HGF	c-Met/MAPK/ SCL4A11/SLC26A4	increased expression of bicarbonate and anion transporter proteins	improved tear film composition	[26]

Abbreviations: mesenchymal stem cells (MSC); dry eye disease (DED); epidermal growth factor (EGF); epidermal growth factor receptor (EGFR); mitogen-activated protein kinase (MAPK); extracellular signal-regulated kinases (ERK); aquaporin 4 and 5 (AQP4 and AQP5), mucin 5AC (MUC5AC), amylase (AMYL), lipoprotein lipase (LIPF); basic fibroblast growth factor (bFGF); fibroblast growth factor receptor (FGFR); serine protease inhibitor kazal type 1 (SPINK1); platelet-derived growth factor A (PDGFA); vascular endothelial growth factors A (VEGFA); Phosphatidylinositol 3-kinase (PI3K); calcium-activated chloride channel regulator 1 (CLCA1); anterior gradient 2 (AGR2); solute carrier family 4 member 11 (SLC4A11); solute carrier family 26 member 4 (SLC26A4).

## 5. MSC-Based Suppression of Detrimental Immune Response in the Eyes of DED Patients

In addition to their capacity to attenuate tear hyperosmolarity-induced pathological changes, MSCs are also able to inhibit immune cell-driven inflammation in the eyes of DED patients, preventing progression and aggravation of DED [Table 2] [20]. MSCs, in a paracrine manner, through the activity of MSC-derived immunomodulatory factors, suppress the proliferation of inflammatory Th1 and Th17 cells, induce the generation and expansion of immunosuppressive Tregs, and create an anti-inflammatory environment in the injured and inflamed eyes of DED patients, importantly contributing to the alleviation of DED-related signs and symptoms [13,15,20].

MSC-derived IL-1Ra and MSC-derived soluble TNF receptor (sTNFR) suppress IL-1β and TNF-α-driven recruitment of circulating inflammatory cells. When MSC-derived IL-1Ra binds to the IL-1 receptor (IL-1R) on endothelial cells in the eyes of DED patients, the binding of IL-1β to IL-1R is blocked and pro-inflammatory signals elicited from IL-1R are stopped [27]. Similarly, MSC-derived sTNFR acts as an inhibitor of inflammatory TNF-α, preventing its binding to TNFR on endothelial cells [28]. In this way, various pro-inflammatory events, initiated by IL-1β:IL-1R or TNF:TNFR binding, including the synthesis and releases of T cell-attracting chemokines, enhanced influx of leucocytes and post-translational modifications of ion channels in inflamed eyes of DED patients, are inhibited by MSC-derived IL-1Ra and sTNFR [27,28]. In line with these findings, the derived Multiple Allogeneic Proteins Paracrine Signaling (d-MAPPS) ophthalmic solution, which contains AF-MSC-derived IL-1Ra and sTNFR, managed to alleviate eye inflammation, support tear stability, and prevent ocular surface epithelial damage, crucially contributing to the enhanced repair and regeneration of ocular surface epithelial barrier in DED patients. Eye discomfort and pain were successfully reduced in 131 DED patients who received d-MAPPS ophthalmic solution. They experienced a substantial improvement in all DED-related symptoms, as evidenced by the significant drop in VAS and SPEED scores 3 months after starting treatment, with the maximum improvement seen after a year of treatment, showing its long-term positive impact on reducing eye symptoms in patients with DED [29].

In addition to IL-1Ra and sTNFR, MSC-derived TGF-β also importantly contributed to the beneficial effects of MSCs in the attenuation of T cell-driven inflammatory response in DED patients [20,30]. MSC-sourced TGF-β blocks the activation of the JAK/STAT signaling pathway in IFN-γ-producing Th1 and IL-17-producing Th17 cells, causing cell cycle arrest at the G0/G1 phase [31]. In this way, MSC-derived TGF-β inhibited the proliferation and expansion of inflammatory Th1 and Th17 lymphocytes, significantly reducing their number in inflamed eyes of DED patients [30,31]. In line with these findings, Ma and colleagues demonstrated that exosomes obtained from UC-MSCs (UC-MSC-Exos) inhibited the proliferation of Th17 cells by causing cell cycle arrest at the G0/G1 phase and facilitated the growth of Tregs by enhancing Foxp3 expression in naïve CD4+ T cells. This increase in the Treg/Th17 ratio correlated with a reduction in the production of inflammatory cytokines (IFN-γ, TNF-α, IL-6, IL-17A, and IL-17F) and an elevation in the secretion of immunosuppressive TGF-β and IL-10 in T cells that were primed with UC-MSC-Exos [32]. Based on these encouraging findings, a clinical trial (NCT04213248) is currently recruiting participants to explore the therapeutic effects of UC-MSC-Exos on alleviating DED-related symptoms in patients with ocular graft-versus-host disease (oGVHD). According to the study protocol, patients will first use artificial tears for 14 days and, afterward, will receive UC-MSC-exosome eye drops (10 μg/drop, administered four times daily) for two weeks. Patients will be monitored for 12 weeks and evaluations will include the changes in ocular surface disease index, conjunctival redness scores, tear secretion, tear break-up time, ocular surface staining, best-corrected visual acuity, and tear meniscus height. Initial results from this trial are expected within the next two years.

In a similar manner as TGF-β, MSC-derived IL-10 also importantly contributes to the creation of an immunosuppressive environment in the inflamed eyes of DED patients [20,31,33]. IL-10 promotes the formation of tolerogenic DCs, which subsequently interact with naïve CD4+ T cells to facilitate their differentiation into FoxP3+ Tregs, contributing to the generation of an immunosuppressive environment in the inflamed eyes of DED patients [31]. However, chronic eye inflammation attenuates Treg-driven immunosuppression [4]. Continuous antigen-driven priming of T cell receptors leads to the phosphorylation and activation of PKB/Akt and the mammalian target of rapamycin (mTOR) in resting Tregs. The activation of the PKB/mTOR pathways alters the immunoregulatory properties of Tregs, causing them to shift into a pro-inflammatory Th17-like phenotype. This transformation results in increased production of inflammatory cytokines, specifically IL-17 and IL-22 [34]. MSC-derived IDO reduces tryptophan levels in the inflamed environment, activating the general control nonderepressible 2 (GCN2) kinase. This activation inhibits the PKB/mTOR2 signaling pathway in Tregs, preventing their conversion into inflammatory Th17 cells [31]. Thus, MSCs, in an IDO-dependent manner, suppress the formation of Th17 cells and promote the expansion of immunosuppressive Tregs in the inflamed eyes of DED patients, aiding in the Treg-mediated reduction in Th17 cell-driven inflammation [29]. Exosomes that were obtained from labial-gland-derived MSCs (LG-MSC-Exos) managed to attenuate DED-related signs and symptoms in experimental mice. LG-MSC-Exos reduced serum levels of Th17-related inflammatory cytokines (IL-6 and IL-17), elevated the levels of the immunosuppressive cytokine TGF-β, reduced the presence of Th17 cells, increased the expansion of Tregs, and improved tear secretion in experimental animals [35].

**Table 2:** Molecular mechanisms responsible for the beneficial effects of MSC-derived immunomodulatory factors in the attenuation of immune cell-driven eye inflammation.

MSC-Derived Factor	Target Cell(s)	Molecular Mechanisms Responsible for MSC-Based Immunoregulation	MSC-Dependent Beneficial Effects	Ref. No
IL-1Ra	endothelial cells	binding of IL-1β to IL-1R is blocked; pro-inflammatory signals elicited from IL-1R are stopped	reduced synthesis and release of T cell-attracting chemokines; reduced post-translational modifications of ion channels	[27]
sTNFR	endothelial cells	pro-inflammatory signals elicited by TNF-α are stopped	decreased influx of circulating leucocytes in inflamed eyes	[28]
IDO	Tregs	inhibition of PKB/mTOR2 signaling pathway	prevented trans-differentiation of Tregs in inflammatory Th1 cells	[29]
TGF-β	Th1 and Th17 cells	suppression of JAK/STAT signaling; G0/G1 cell cycle arrest	reduced number of activated, inflammatory T lymphocytes in the eyes of DED patients	[31]
IL-10	DCs	increased generation of tolerogenic DCs that favor expansion of immunosuppressive Tregs	generation of immunosuppressive microenvironment in the inflamed eyes	[31]

Abbreviations: interleukin 1 receptor antagonist (IL-1Ra); interleukin 1 beta (IL-1β); interleukin 1 receptor (IL-1R); soluble receptor of tumor necrosis factor alpha (sTNFR); tumor necrosis factor alpha (TNF-α); indoleamine 2,3-dioxygenase (IDO); protein kinase B (PKB); mammalian target of rapamycin (mTOR); T regulatory cells (Tregs); transforming growth factor beta (TGF-β); Janus kinase/signal transducers and activators of transcription (JAK/STAT); interleukin-10 (IL-10); dendritic cells (DCs).

## 6. Trophic Effects of MSCs in the Inflamed Eyes of DED Patients

In addition to their immunosuppressive effects, MSCs produce a large number of growth factors (HGF, bFGF, TGF-β, insulin-like growth factor (IGF),) and pro-angiogenic molecules, which could prevent apoptosis and improve the viability of injured CECs, ConECs, GCs, and ACs in the eyes of DED patients [20,22,33].

MSC-derived HGF is capable of supporting the survival of injured cells through the modulation of Bcl-2 family proteins [26,31,33]. When MSC-derived HGF interacts with its receptor c-Met on injured cells, it triggers a cascade of intracellular signaling pathways that lead to the up-regulation of Bcl-2 and Bcl-XL proteins, which promote survival and inhibit apoptosis. HGF-dependent activation of the c-Met receptor leads to the recruitment and phosphorylation of adaptor protein Grb2. Grb2, in turn, activates Ras, which initiates a signaling cascade involving Raf, MEK, and ERK. Activated ERK translocates to the nucleus and regulates the expression of pro-survival Bcl-2 and Bcl-XL genes. In the cytoplasm of injured cells, HGF:c-Met signaling can also activate the signal transducer and activator of transcription (STAT) proteins, which translocate to the nucleus and enhance the expression of Bcl-2 and Bcl-XL genes [26,31].

**Figure 4:**
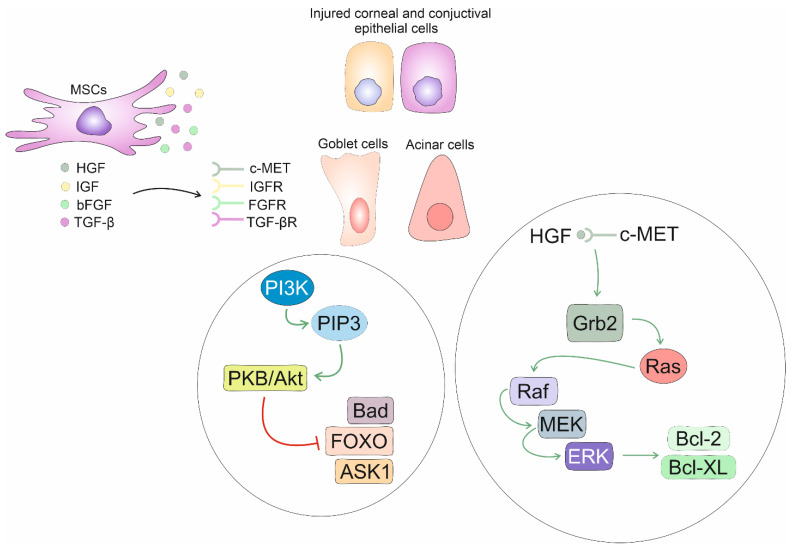
Molecular mechanisms responsible for the trophic effects of MSCs in the inflamed eyes of DED patients. MSC-derived HGF, IGF, bFGF, and TGF-β bind to their receptors (c-Met, IGFR, FGFR, TGFβR) on injured corneal and conjuctival epithelial cells, goblet cells, and acinar cells of lacrimal glands and activate PI3K that generates PIP3, which in turn activates PKB/Akt kinase. Akt phosphorylates inhibit several pro-apoptotic factors (Bad, FOXO, ASK1), preventing apoptosis of injured cells. MSC-derived HGF modulates expression of pro-survival Bcl-2 and Bcl-XL proteins, as well. HGF-dependent activation of c-Met receptor leads to the recruitment and phosphorylation of adaptor protein Grb2. Grb2, in turn, activates Ras, which initiates a signaling cascade involving Raf, MEK, and ERK. Activated ERK translocates to the nucleus and regulates the expression of pro-survival Bcl-2 and Bcl-XL genes, importantly contributing to the improved viability of injured cells in the eyes of DED patients.

MSC-sourced bFGF acts synergistically with MSC-derived HGF, IGF, and TGF-β to stimulate protein kinase B (PKB/Akt) kinase activity in injured CECs, ConECs, GCs, and ACs, promoting their survival [14,22,36]. The combined action of these factors helps to enhance the regenerative capacity of MSCs and facilitate the MSC-driven healing process in inflamed eyes of DED patients [22,36]. Upon binding to their receptors on injured cells, these growth factors activate phosphatidylinositol 3-kinase (PI3K) that generates phosphatidylinositol (3,4,5)-trisphosphate (PIP3), which in turn activates PKB/Akt kinase [22]. Akt phosphorylates and inhibits several pro-apoptotic factors (Bcl-2-associated death promoter (Bad), Forkhead box O (FOXO), ASK1), preventing apoptosis of injured cells [36] [Figure 4]. Akt phosphorylates Bad protein, leading to its sequestration in the cytoplasm. Also, phosphorylated Bad is unable to bind and inhibit anti-apoptotic Bcl-2 and Bcl-XL proteins, thereby preventing the initiation of the intrinsic apoptotic pathway. Akt also phosphorylates and inhibits the FOXO family of pro-apoptotic transcription factors, which regulate the expression of genes involved in cell cycle arrest and apoptosis [22,36]. Akt can directly phosphorylate and inhibit ASK1 kinase, which is a key regulator of stress-induced mitochondrial apoptosis. By inhibiting ASK1, Akt prevents the activation of the stress-induced apoptotic pathway in injured CECs, ConECs, GCs, and ACs, promoting their survival [36].

In the inflamed eyes of DED patients, MSCs provide vascular support to injured CECs, ConECs, GCs, and ACs through the activity of MSC-sourced pro-angiogenic factors (VEGF, angiopoietins, HGF, bFGF, and PDGF) [20,25,37]. MSC-derived VEGF promotes angiogenesis, enhancing blood flow to the injured area and providing nutrients for cell regeneration. VEGF promotes the formation of new blood vessels by enhancing endothelial cell survival and increasing vascular permeability [25]. In addition to VEGF, MSC-derived Ang-1 and Ang-2 are crucial regulators of angiogenesis, influencing the formation, stabilization, and remodeling of blood vessels. Ang-1 binds to the Tie2 receptor on endothelial cells, promoting cell survival and stability. This interaction enhances the integrity of blood vessels and prevents excessive permeability, which is vital for maintaining vascular homeostasis. MSC-sourced Ang-1 is essential for the maturation of newly formed blood vessels. It recruits pericytes to the developing vasculature, contributing to vessel stabilization and maturation. MSC-sourced Ang-2 acts as a competitive antagonist to Ang-1 for the Tie2 receptor, leading to the destabilization of existing blood vessels. In this way, MSC-derived Ang-2 prevents excessive or uncontrolled vessel growth, which is crucial in maintaining balance during the wound-healing process. In inflamed lacrimal glands, MSC-derived Ang-2 increases vascular permeability, allowing plasma proteins and immune cells to migrate into surrounding tissues [20,37]. Additionally, in the presence of VEGF, MSC-derived Ang-2 enhances endothelial cell migration and proliferation [20,25,37]. It facilitates the angiogenic switch by allowing endothelial cells to respond to other growth factors, enabling an optimal supply of nutrients to injured ACs. The balance between MSC-sourced Ang-1 and Ang-2 is crucial for normal angiogenesis in inflamed lacrimal glands of DED patients [20,37].

HGF, PDGF, and bFGF are also potent pro-angiogenic factors released by MSCs. MSC-sourced HGF and bFGF stimulate the proliferation and migration of endothelial cells, contributing to new capillary formation, while MSC-derived PDGF supports the proliferation and migration of smooth muscle cells and pericytes, stabilizing the integrity of new blood vessels [24,26,37]. PDGF binds to and activates PDGF receptors on target cells. This activation initiates a cascade of intracellular signaling pathways, including the Ras-MAPK and PI3K-Akt pathways, promoting cellular responses associated with cell growth and survival. PDGF plays a crucial role in recruiting pericytes to nascent blood vessels. Once recruited, pericytes secrete PDGF, Ang-1, VEGF, and Sphingosine-1-phosphate (S1P) that promote the maturation and stabilization of blood vessels. This stabilization is essential for maintaining vessel integrity and preventing excessive permeability of newly generated blood vessels. In larger blood vessels, PDGF also plays a role in the recruitment and proliferation of smooth muscle cells, which are essential for the structural integrity of blood vessels [26]. This contribution is particularly important during the remodeling phase of angiogenesis in injured lacrimal glands of DED patients [26,37].

## 7. Conclusions

**Figure 5:**
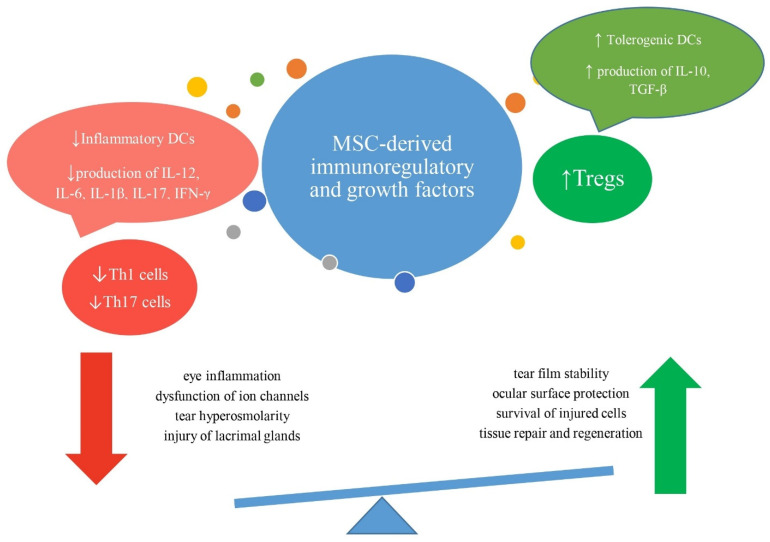
Beneficial effects of mesenchymal stem cell (MSC)-dependent attenuation of tear hyperosmolarity-induced pathological changes in the inflamed eyes. MSC-derived immunomodulatory factors inhibit activation of inflammatory dendritic cells (DCs) and induce generation of immunosuppressive, tolerogenic DCs, attenuate production of inflammatory cytokines (IL-12, IL-6, IL-1β, IL-17, IFN-γ), and enhance production of immunosuppressive cytokines (transforming growth factor beta (TGF-β) and IL-10), reducing number of inflammatory Th1 and Th17 cells and promoting expansion of immunosuppressive T regulatory cells (Tregs) in the inflamed eyes. In this way, MSC-derived immunomodulatory factors inhibit inflammation-driven dysfunction of ion channels, attenuate tear hyperosmolarity, and reduce injury of lacrimal glands. MSC-sourced growth factors improve tear film stability, support the survival of injured cells, enhance ocular surface protection, and repair and regenerate injured tissues.

MSCs play a pivotal role in mitigating the effects of DED through their multifaceted mechanisms. By releasing growth factors such as EGF, bFGF, and HGF, MSCs enhance the secretion of essential tear film components, significantly improving tear quality and quantity. This was crucial in alleviating tear hyperosmolarity, promoting ocular surface protection, and ensuring tear film stability. Furthermore, MSC-derived IL-1Ra, IDO, HGF, TGF-β, and IL-10 exhibited a robust immunomodulatory capacity, effectively suppressing inflammatory responses by inhibiting activation of inflammatory DCs, Th1, and Th17 lymphocytes and promoting the expansion of immunosuppressive Tregs. By suppressing harmful immune responses, MSCs prevented inflammation-driven post-translation modification and dysfunction of ion channels, attenuating tear hyperosmolarity-induced pathological changes in the eyes of DED patients [Figure 5]. In addition to their immunosuppressive activity, MSCs provided trophic support to damaged CECs, GCs, and ACs in the inflamed eyes, importantly contributing to enhanced tissue repair and regeneration. In the end, it should be noted that although results obtained in preclinical and pilot clinical studies demonstrated that MSC-based therapy successfully addressed the most severe DED-related conditions and complications, upcoming large clinical trials should confirm these findings in clinical settings before MSCs could be widely offered as new therapeutic agents for the treatment of DED and other inflammatory eye diseases.

## Data Availability

The data that are discussed in this article are presented in cited studies.
